# Adherence to Treatment, Quality of Life, and Level of Knowledge in Patients on Anticoagulant Therapy with Vitamin K Antagonists

**DOI:** 10.3390/healthcare14081042

**Published:** 2026-04-15

**Authors:** Adolfo Romero-Arana, Nerea Romero-Sibajas, Juan Gómez-Salgado, María Isabel Ruiz-Moreno, Víctor Manuel Cotta-Luque, Lucía Rojas-Suárez, Luis El Khoury-Moreno, Julio Torrejón-Martínez, Adolfo Romero-Ruiz

**Affiliations:** 1 Fundación Jiménez Díaz School of Nursing—Autonomous University of Madrid, Health Research Institute-Fundación Jiménez Díaz University Hospital, Autonomous University of Madrid (IIS-FJD, UAM), 28040 Madrid, Spain; 2 Universidad Alfonso X Mare Nostrum, Camino de la Térmica 90, 29004 Málaga, Spain; mrisblrz@uax.es; 3 Pediatric Nutrition Nursing Consultation, Hospital Universitario La Paz, Paseo de la Castellana 261, Fuencarral-El Pardo, 28046 Madrid, Spain; nerearomero95@gmail.com; 4 Department of Sociology, Social Work and Public Health, Faculty of Labour Sciences, University of Huelva, 21007 Huelva, Spain; salgado@uhu.es; 5 Safety and Health Postgraduate Programme, Universidad Espíritu Santo, Guayaquil 092301, Ecuador; 6 Distrito Sanitario Málaga-Guadalhorce, Andalusian Health System, 29009 Málaga, Spainlucia.rojas.sspa@juntadeandalucia.es (L.R.-S.); 7 Department of Stomatology, Faculty of Odontology, Universidad de Sevilla, 41009 Sevilla, Spain; lelkhoury@us.es (L.E.K.-M.); jtorrejon1@us.es (J.T.-M.); 8 BA-16 Group, Institute of Biomedical Research of Malaga and Platform in Nanomedicine-IBIMA Platform BIONAND, 29590 Málaga, Spain; adolforomeror@uma.es; 9 Department of Nursing and Podiatry, Health Sciences School, University of Málaga, 29071 Málaga, Spain

**Keywords:** oral anticoagulation, self-monitoring, vitamin K antagonists

## Abstract

**Background:** In Spain, the number of patients anticoagulated with vitamin K antagonists (VKAs) is high. Among them, poor adherence is common, which may be justified by a low level of knowledge, and could affect their quality of life. We analyzed treatment adherence, health-related quality of life, and knowledge level about treatment, and evaluated the possible influence of these factors on patients’ time in the therapeutic range while also studying potential differences between patients under routine monitoring or self-monitoring. **Methodology:** A cross-sectional descriptive study was conducted using three validated and cross-culturally adapted questionnaires to study therapeutic adherence, health-related quality of life, and knowledge level about VKA treatment in a sample of anticoagulated patients. Additionally, it was assessed whether they were self-monitoring or not; the Rosendaal Time in Therapeutic Range (TTRr) was also administered for each patient at the time of recruitment. Descriptive analysis of all variables was performed, and a logistic regression model was constructed to evaluate the possible interaction of variables. **Results:** Ninety-eight patients participated and were selected sequentially from those attending the oral anticoagulation clinic at Hospital Universitario Virgen de la Victoria in Malaga. Of these, 39 were men and 59 were women. The mean age of these participants was 60.62 years (SD 11.67). Sixty-six were under conventional monitoring and thirty-two followed the self-monitoring program. The DecaMIRT had a mean score of 39.22 (SD 8.57), the SF-12 mean score was 31.73 (SD 6.21), and the knowledge questionnaire’s was 14.2 (SD 2.6). The mean TTRr value was 63.88 (SD 22.99). Self-monitored patients showed better results in DECAMirt and knowledge. **Discussion:** Overall, patients included in the sample presented satisfactory values in these three questionnaires, which seems to indicate that this was a treatment-compliant group with a correct quality of life, and adequately informed about their treatment. **Conclusions:** The work of nurses responsible for these aspects appears crucial in achieving these results. We aim to extend this study by focusing on groups with poorer results to design specific activities that allow for improvement in care and, as much as possible, homogenize outcomes. For this purpose, we intend to use all available tools, including those derived from the use of health-oriented artificial intelligence.

## 1. Introduction

Anticoagulant therapy has been, for many years, a widely used therapeutic strategy in patients with a specific profile: either at risk of suffering a cardioembolic stroke (as atrial fibrillation patients) or at risk of second thromboembolic events (after a first event or after the implantation of a metallic cardiac valve) [1,2,3], even though it is known that these treatments carry a series of associated risks that require controlled management, especially in elderly patients [4].

The classic approach has been with anti-vitamin K drugs (VKAs), although recently there has been a shift in this regard, specifically since the emergence of direct-acting anticoagulants (DOACs) currently indicated for AF and the prevention of secondary thrombotic events, a treatment sometimes preferred by patients [5,6].

However, in some cases this is not possible, and they must follow prophylaxis for thrombotic events through therapy with VKAs, as is the case with mechanical valve prostheses or antiphospholipid syndrome and arterial thrombosis [7,8].

The analytical control of patients undergoing treatment with VKAs is currently carried out through the determination of the international normalized ratio (INR) with portable coagulometers, which use dry chemistry procedures to analyze a small amount of capillary blood and allow obtaining prothrombin times (PTs) similar to those obtained in the laboratory. There are different models with similar characteristics that allow good results expressed in INR to be obtained [9].

The next parameter considered concurrently in the monitoring of these patients, intimately related to the measurement of INR, is time in the therapeutic range as measured by the Rosendaal Therapeutic Range Time or TTRr [10]. Maintenance within the range between analytical determinations is established in a “theoretical” manner, primarily using data obtained from the pharmacokinetics of acenocoumarol and clinical experience and management [11,12].

One of the factors to consider in these patients is that performing INR checks requires travelling to healthcare facilities, an issue that, under certain circumstances, can have a significant impact on the normal life of patients, which can affect their health-related quality of life (HRQoL) [13]. Although there are ongoing strategies to mitigate this problem—such as self-monitoring, which has been shown to be very effective [14], sometimes the special characteristics of these patients, difficulty in accessing these specific programs, or their own choice make it impossible to use this method, so travel must occur.

In addition, patients undergoing treatment with oral anticoagulants (VKAs) require specific training at the start of treatment, which, at our center, is provided by specialized nurses.

This particular aspect, along with the skills and knowledge necessary to carry out proper adherence and control of the treatment, in addition to clinical characteristics present in these patients, could affect, as mentioned, HRQoL. Therefore, we proposed and designed a study that examines the relationship between these factors, which are considered relevant in this patient group. 

This study was designed to evaluate these previously described variables— time in therapeutic range of a sample of patients treated with vitamin K antagonists, their degree of adherence to treatment, their level of knowledge about it, and their HRQoL—as three different factors that might be influenced in the follow-up of VKA-treated patients. Also, was separately analyzed in those patients who were self-monitoring, a procedure that was reported years ago to have a relevant impact on HRQoL [15].

## 2. Materials and Methods

### 2.1. Study Design and Instruments

A cross-sectional descriptive study was conducted using three validated and cross-culturally adapted questionnaires to study therapeutic adherence: the DECAMirt [15], HRQoL (SF-12) [16], and knowledge level about VKA treatment (Knowledge Questionnaire on VKA) [17].

The DECAMirt test measures the management of the treatment plan, in 10 items, from knowledge about the process to how to respond in an emergency. Scores range from 10 (minimum) to 50 (higher).

HRQoL (SF-12) (1994, 2003 Health Assessment Lab, Medical Outcomes Trust, and QualityMetric Incorporated, Lincoln, RI) is represented by 12 items that measure the quality of life in 8 dimensions: Physical Function, Body Pain, General Health, Vitality, Social Function, Emotional Role and Mental Health. Scores range from 0 to 100.

The Knowledge Questionnaire on VKA is a 20-item questionnaire, with four answers for each item, but only one correct answer. Scores range from 0 (minimum) to 20.

### 2.2. Population and Sample

A sample was selected from the population of patients anticoagulated with VKAs in the Málaga-Guadalhorce health area, which includes the Oral Anticoagulation Clinic of the Hospital Universitario Virgen de la Victoria in Málaga, 18 Health Centers, and 9 consulting offices. Approximately 1200 anticoagulated patients were treated in this area. The sample size calculation was performed using the statistical program JAMOVI, version 2.7.6, with the power analysis with linear model extension module. It was set up for mean comparison, with a confidence level of 95% and a margin of error of 10%, resulting in 89 patients. The sample was overestimated by 10% to account for possible losses, finally including 98 subjects.

Sampling was incidental, sequential, and non-randomized, offering participation to patients from the start of the study (May 2023) until the necessary sample size was achieved (February 2025).

### 2.3. Study Variables

Sociodemographic variables (age, sex), type of control (conventional or self-control), reason for anticoagulation, and TRTr value at the time of study recruitment were analyzed. Age was categorized to perform linear regression analysis.

TTR represents the percentage of time in which the INR remained in the target range across time. At first glance, TTR might simply be the number of INR values in the target range (numerator) over the total number of INR values measured (denominator); that is, the fraction of INRs in the target range. However, linear interpolation methods were employed more often, which offered an estimation of each patient’s anticoagulation status between INR determinations [18].

On the other hand, continuous values were employed (score data) in the other instruments.

### 2.4. Statistical Analysis

Measures of central tendency and dispersion (means and standard deviations) were analyzed, which confirmed the normal distribution of data, so parametric tests were used.

Multivariate analysis (MANCOVA) was performed. Those values that showed statistical significance were analyzed with pairwise tests, such as Student’s *t* test, for confirmation. Additionally, data were confirmed using one-way ANOVA to observe differences between the means of both groups. Finally, a logistic regression model was constructed to evaluate the possible interaction of the variables, taking as dependent variables sex, age (codified) and self-controlled/not self-controlled patients. 

### 2.5. Ethical Aspects

The researchers strictly adhered to the provisions of this protocol, fully completing the data collection notebook sheets. This study was conducted in accordance with the standards of Good Clinical Practice, the ethical principles of the Declaration of Helsinki “75th WMA General Assembly, Helsinki, Finland, October 2024”, and the current legislation in Spain under Law 14/2007 of July 3, on Biomedical Research. The processing, communication, and transfer of personal data of all participating patients complied with the provisions of Regulation (EU) 2016/679 of the European Parliament and of the Council of 27 April 2016 on Data Protection (GDPR) and Organic Law 3/2018 of December 5, on the Protection of Personal Data and guarantee of digital rights.

This project was approved for implementation by the Provincial Research Ethics Committee of Malaga, code TAO-005, on 24 February 2022.

## 3. Results

A total of 98 patients participated. Of these, 39 were men and 59 were women. The mean age of these participants was 60.62 years (SD 11.67). Sixty-six patients were under conventional follow-up, and thirty-two followed the self-management (AC) program. The mean TTRr value was 63.88 (SD 22.99). The DecaMIRT had a mean score of 39.22 (SD 8.57), the SF-12 of 31.73 (SD 6.21), and the knowledge test of 14.2 (SD 2.6) (Table 1). Women were younger in all groups (Table 1).

Regarding TTRr, women showed higher values in all subgroups, with the non-self-controlled subgroup having the highest values. On the other hand, the DecaMIRT was almost identical in men and women in the grouped values, although it was higher in self-controlled women, with statistically significant differences (*p*, 0.018) (Table 2).

The SF-12 was higher in the self-controlled group, especially in men. When adjusting for sex and AC, differences in the questionnaire were statistically significant (*p* = 0.008) (Table 2).

Knowledge was higher in the self-controlled group, especially among men. In nonself-controlled women, the score was below average, and this difference was one and a half points compared to self-controlled women, with statistically significant differences (*p* < 0.001) (Table 1).

In the multivariate analysis, values that showed statistical significance were analyzed with pairwise tests, such as Student’s *t* test, to confirm the significant results.

Regarding the MANCOVA, to evaluate possible differences between all the studied factors, patients in AC had a higher level of knowledge (*p* < 0.001) and there was a higher number of women in the AC program (*p* = 0.008). The differences between the self-control group and those not belonging to it were evaluated. Significant differences were found in the values of the DECAMirt test and the knowledge test on oral anticoagulation. These data were confirmed using Student’s *t* test to observe the differences between the means of both groups. (*p* = 0.005 and *p* = 0.002, respectively) (Table 3 and Table 4).

Significant logistic regression models were based on age, although the values that could explain these differences had very low values (DECAMirt, 0.2715, and Knowledge, 0.264), and the one built for AC was the dependent variable. This was influenced by sex and level of knowledge. Age explains more than 60% of the model—the older the age, female sex, and higher level of knowledge, the better the results. In some way, these variables acted as “protective factors”, yielding higher scores on knowledge tests in self-controlled patients and in women.

## 4. Discussion

As previously mentioned, a large number of anticoagulated patients require the use of specific strategies to ensure that their clinical care does not hinder their performance in daily activities. This is currently important due to the significant increase in cases among the young population related to the COVID-19 pandemic [19], a demographic profile that works and/or studies and differs from the previously known typical patient, usually over 65 years old with comorbidities and sometimes a certain degree of dependency [20].

We obtained some interesting results derived from the TTRr of the study population (63.88), which were near to the mean results of our health system (69.1) [21], indicating an adequate level of adherence and self-perceived health and an “a priori” good knowledge level of the treatment (Table 1). 

The present study topic has been previously investigated, although with other instruments. Regarding health-related quality of life, a similar study conducted in 2005 revealed that only 14% of patients had good anticoagulation control, while in the present study the TTRr was 63.88, very close to the established minimum threshold of 65. This parameter was not commonly used at the time of the referenced study [22]. Fifty percent of patients reported adequate adherence, which was significantly associated with good anticoagulation control (*p* = 0.01). Thirty-seven percent of participants had good knowledge about anticoagulation, and 19% stated that ACO had a negative impact on their quality of life [22]. The results obtained in this study provide better data in all three areas, and there were twice as many patients.

On the other hand, a study on health-related quality of life conducted in Brazil with a sample of 172 patients revealed that women, elderly individuals, and patients diagnosed with atrial fibrillation and with less than one year of medication use had worse HRQoL assessments [23]. These data differ from those obtained in the present study, as men showed better results in the Auto-Control (AC) subgroup, with women achieving better data both in the total aggregate and in the non-self-controlled subgroup; on the other hand, age does not show statistically significant differences in any of these cases. Certain aspects, such as work performance, which is typically more common in men, may have influenced these results [24], although we do not disregard that the analyzed sample, although significant, was not large.

Regarding adherence to treatment with these drugs, the interesting study by Abdou et al. highlighted that younger patients and men are more likely to discontinue treatment and not comply with VKA therapy, while patients with higher thrombotic risk tend to continue treatment longer and adhere to it better [25]. According to the presented results, men also showed worse data, especially those following the AC program (37.87 vs. 40.78), which aligns with previously available information.

The subgroup that does show better results is that of self-controlled patients, which had higher scores in all categories except TTRr in women, which suggests that the actions implemented in the training and monitoring of these patients were highly effective [14].

Finally, the level of knowledge was higher in men in the two analyzed subgroups (14.64 vs. 13.91), although in both cases the cut-off point, set at 12 points, was exceeded. Previous studies have emphasized the importance of understanding the treatment well and the risk of having insufficient knowledge, which can jeopardize safety and effectiveness [26]. Our values were lower than those of the self-controlled population in our environment, suggesting the need to use corrective dynamics in this regard, which can help avoid the increased risk of complications [14]. In this way, there is room for services to address barriers in each area to improve compliance with VKA therapy, from optimizing opportunities by simplifying dosing regimens to increasing motivation by dispelling myths surrounding VKAs [25]. In this sense, apart from traditional methods, with specific training sessions the use of artificial intelligence could be a powerful tool to better understand the problems associated with the level of knowledge [26,27,28].

As limitations of this study, we propose those inherent to the chosen design, which prevented causal inference. Moreover, self-reported questionnaires, which may introduce reporting bias, and non-probabilistic sampling, might have affected generalizability. Finally, there might have been some potential confounding variables not controlled in the analysis, although we performed robust statistical analysis in order to minimize them. 

Additionally, having conducted this study in a specific area with a relatively small number of participants could have compromised the external validity of the results. To address this, we aim to conduct a similar, but multicenter, study, and plan actions involving interventions that can demonstrate their effectiveness in these patients to mitigate this bias.

## 5. Conclusions

Results showed that, overall, patients included in the sample achieved satisfactory scores on all three questionnaires, which seems to indicate that patients in this group adhered to their treatment, enjoyed a good quality of life and were well informed about their treatment. We believe that the work of the nurses responsible for these aspects was crucial to achieving these results. 

Additionally, it was observed that patients who followed AC programs generally presented better outcomes than those who did not, so it seems that adapting the actions carried out with them would be a good strategy to improve, at all levels, the aspects measured in our study. This fact, linked to the advancement of new technologies—such as the development of mobile applications, the digitization of health records and their use, and the development of artificial intelligence (IA)—as we are currently investigating opens the possibility of expanding the horizon of self-managed patient groups. This is especially significant considering the previously described circumstances: there are more young patients requiring treatment with anticoagulants, so older people have more difficulty getting these treatments handled, and IA models may help them, together with health professionals.

Therefore, it is necessary to update knowledge on the subject and investigate the behavior of these possible new profiles in patients. Along with this, it is proposed to expand knowledge in this regard, including the development of new tools [29] to promote self-care and improve the quality of life and care of these patients.

## Figures and Tables

**Table 1:** Descriptive analysis and questionnaire scores.

	WOMEN	MEN	AC WOMEN	AC MEN	No AC WOMEN	No AC MEN	TOTAL
Subjects	39	59	9	23	30	36	98
Age	62.0 (11.98)	60.0 (11.55)	61.83 (10.68)	57.47 (10.05)	62.04 (12.48)	60.97 (12.26)	60.62 (11.67)
x̄ (SD) TTRr	61.73 (22.68)	65.34 (23.17)	61.7 (24.1)	62.9 (21.7)	61.66 (22.7)	67.06 (24.34)	63.88 (22.39)
x̄ (SD) DECAMIRT	38.56 (8.41)	38.61 (8.67)	37.87 (10.07)	40.78 (8.15)	37.37 (8.64)	37.06 (9.65)	38.46 (9.01)
x̄ (SD) SF-12	30.66 (5.21)	32.29 (6.82)	33.56 (7.52)	30.85 (5.6)	29.73 (4.44)	33.26 (7.48)	31.36 (6.76)
x̄ (SD) Knowledge	14.64 (2.45)	13.91 (2.65)	16.44 (1.87)	14.76 (2.87)	14.1 (2.37)	13.26 (2.93)	14.09 (2.83)

**Table 2:** MANCOVA analysis. * Name of the variable in the database, means TTRr.

Univariate Tests						
	Dependent Variable *	Sum of Squares	gl	Root Mean Square	F	*p*
**“SEX”**	“TRT 1” *	26.668	1	26.668	0.0465	0.83
	**“DECAMirt”**	2.285	1	2.285	0.0359	0.85
	**“SF12”**	80.208	1	80.208	2.3102	0.133
	**“ACO Knowledge”**	22.16	1	22.16	3.951	0.05
**“AC”**	“TRT 1” *	440.596	1	440.596	0.768	0.384
	**“DECAMirt”**	372.926	1	372.926	5.8557	0.018
	**“SF12”**	0.496	1	0.496	0.0143	0.905
	**“ACO Knowledge”**	66.501	1	66.501	11.8568	**<** **0.001**
**“SEX” ✻ “AC”**	“TRT 1” *	57.686	1	57.686	0.1006	0.752
	**“DECAMirt”**	2.845	1	2.845	0.0447	0.833
	**“SF12”**	253.739	1	253.739	7.3083	**0.008**
	**“ACO Knowledge”**	6.677	1	6.677	1.1904	0.279
**“AGE”**	“TRT 1” *	568.668	1	568.668	0.9913	0.323
	**“DECAMirt”**	192.872	1	192.872	3.0285	0.086
	**“SF12”**	27.544	1	27.544	0.7933	0.376
	**“ACO Knowledge”**	5.995	1	5.995	1.0688	0.305
**Residuals**	“TRT 1” *	43,024.196	75	573.656		
	**“DECAMirt”**	4776.459	75	63.686		
	**“SF12”**	2603.963	75	34.72		
	**“ACO Knowledge”**	420.655	75	5.609		

**Table 3:** Student’s *t* tests. * Name of the variable in the database, means TTRr.

Independent Samples *t* Test						
		Statistic	gl	*p*	Mean Difference	SE of the Difference
“TRT 1” *	**Student’s t**	0.381	95.0	0.648	1.879	4.936
**“Score** **DECAMirt”**	**Student’s t**	−2.600	95.0	0.005	−4.615	1.775
**“SF12” (2)**	**Student’s t**	−0.686	95.0	0.247	−0.911	1.329
**“Knowledge** **ACO”**	**Student’s t**	−3.034	95.0	0.002	−1.616	0.533
**Note: H_a_ μ_NO_ < μ_YES_**						

**Table 4:** Mean scores.

Group Descriptors					
	“AC”	N	Mean	SD	SE
**“Score** **DECAMirt”**	**NO**	64	37.2	9.12	1.140
	**YES**	33	41.8	6.31	1.099
**“SF12”**	**NO**	64	31.4	6.54	0.817
	**YES**	33	32.3	5.49	0.955
**“Knowledge** **ACO”**	**NO**	64	13.7	2.69	0.336
	**YES**	33	15.3	2.02	0.352

## Data Availability

Data are fully available upon request to the corresponding author.
